# RNA-Seq-Based Analysis of Cold Shock Response in *Thermoanaerobacter tengcongensis*, a Bacterium Harboring a Single Cold Shock Protein Encoding Gene

**DOI:** 10.1371/journal.pone.0093289

**Published:** 2014-03-25

**Authors:** Bo Liu, Yuhong Zhang, Wei Zhang

**Affiliations:** Biotechnology Research Institute, Chinese Academy of Agricultural Sciences, Beijing, China; Imperial College London, United Kingdom

## Abstract

**Background:**

Although cold shock responses and the roles of cold shock proteins in microorganisms containing multiple cold shock protein genes have been well characterized, related studies on bacteria possessing a single cold shock protein gene have not been reported. *Thermoanaerobacter tengcongensis* MB4, a thermophile harboring only one known cold shock protein gene (*TtescpC*), can survive from 50° to 80°C, but has poor natural competence under cold shock at 50°C. We therefore examined cold shock responses and their effect on natural competence in this bacterium.

**Results:**

The transcriptomes of *T. tengcongensis* before and after cold shock were analyzed by RNA-seq and over 1200 differentially expressed genes were successfully identified. These genes were involved in a wide range of biological processes, including modulation of DNA replication, recombination, and repair; energy metabolism; production of cold shock protein; synthesis of branched amino acids and branched-chain fatty acids; and sporulation. RNA-seq analysis also suggested that *T. tengcongensis* initiates cell wall and membrane remodeling processes, flagellar assembly, and sporulation in response to low temperature. Expression profiles of *TtecspC* and failed attempts to produce a *TtecspC* knockout strain confirmed the essential role of *Tte*CspC in the cold shock response, and also suggested a role of this protein in survival at optimum growth temperature. Repression of genes encoding ComEA and ComEC and low energy metabolism levels in cold-shocked cells are the likely basis of poor natural competence at low temperature.

**Conclusion:**

Our study demonstrated changes in global gene expression under cold shock and identified several candidate genes related to cold shock in *T. tengcongensis*. At the same time, the relationship between cold shock response and poor natural competence at low temperature was preliminarily elucidated. These findings provide a foundation for future studies on genetic and molecular mechanisms associated with cold shock and acclimation at low temperature.

## Introduction

Rapid temperature downshifts of more than 15–20°C can cause global alterations in gene expression that result in broad physiological changes collectively referred to as cold shock responses. These include changes in cell wall/membrane composition, protein synthesis rates, energy metabolism, and others [1]. Cold shock responses have been documented in almost all unicellular organisms from thermophiles (such as *Thermus thermophilus* [2], *Thermotoga maritima* [3] and *Thermus* sp. GH5 [4]) to mesophiles (such as *Bordetella bronchiseptica* [5] and *Caulobacter crescentus* [6]) to psychrophiles (such as *Psychromonas arctica* [7] and *Pseudoalteromonas haloplanktis* [8]). The major cold shock protein in *Escherichia coli* (*Eco*CspA) and some of its homologs in other bacterial species play critical roles in cold shock and acclimation of cells, and act as RNA chaperones to destabilize RNA secondary structures at low temperature [9], [10]. The number of *Eco*CspA homologs varies greatly by species. For example, there are nine homologs in *E. coli* (CspA to CspI) [11]; three in *Bacillus subtilis* (CspB, CspC, and CspD), with CspB being the major homolog [12]; and two in *T. thermophilus* HB8, with Csp2 being the major homolog [2]. Cold shock responses of bacteria containing at least two different *csp* genes have been extensively described; however, studies in bacteria harboring a single *csp* gene have not yet been reported.

*Thermoanaerobacter tengcongensis* MB4, a thermophilic and anaerobic bacterium, can survive from 50° to 80°C, with the optimum temperature being 75°C [13]. The full genome of this strain was sequenced in 2002 [14]. Previously, we revealed that *T. tengcongensis* exhibits natural competence and that the transformation efficiency at 50°C is relatively poor compared with 75°C [15]. We postulated that the complicated changes involved in the *T. tengcongensis* cold shock response likely lead to this poor transformation efficiency, although the exact mechanism was not understood. Additionally, in view of the importance of CSPs in the cold shock response of other bacteria, we speculated that the CSPs of *T. tengcongensis* would also be responsible for the cold shock response of this strain. Subsequent analysis of the *T. tengcongensis* genome revealed only one *csp* gene (*TtecspC*) in chromosomal DNA of this strain. Similar genome analysis of multiple microorganisms revealed that possession of a single *csp* gene is a common phenomenon among most thermophilic anaerobes.

Although the proteome and transcriptome of *T. tengcongensis* have been analyzed at various temperatures [16], [17], the effect of cold shock in this species has not been extensively studied. To shed light on the molecular mechanisms of *T. tengcongensis* adaptation to rapid temperature downshifts and of the poor natural competence at low temperature, we performed a global transcriptional analysis of gene expression after cold shock. We found that extensive transcriptional differences occurred after an abrupt decrease in temperature. The results of this study will provide deeper insight into transcriptional regulation of the cold shock response in *T. tengcongensis* and, more broadly, in thermophilic anaerobes.

## Materials and Methods

### Bacterial strains and culture conditions

*T. tengcongensis* MB4 (China General Microbiological Culture Collection Centre, deposit number CGMCC1.2430) was grown in TTE medium as previously described [15].

To investigate the growth of *T. tengcongensis* at 75° versus 50°C, initial cultures were grown at 75°C to an optical density at 600 nm (OD_600_) of 0.6, and then diluted 1/100 in fresh medium, followed by incubation at 75° or 50°C (designated TTE75 and TTE50, respectively). Bacterial growth was monitored from this point by measuring OD_600_.

For the cold shock experiment, cells were grown at 75°C to an OD_600_ of 0.6 as above, and aliquots were then cultured at 50°C for 20, 40, 60, 120, 240, and 360 min (designated TTECS20 to TTECS360). TTECS60 was also designated TTECS, and TTECS01 and TTECS02 were the two biological replicates of TTECS. Bacteria were harvested by centrifugation (8,000× g) at 4°C for 5 min and pellets were washed with 50 mM Tris-HCl (pH 7.8) at 4°C, and then stored at −80°C until RNA extraction. At least two independent cultures were prepared for each time point.

### Total RNA preparation for real-time polymerase chain reaction (PCR) and RNA sequencing (RNA-seq)

Total RNA of *T. tengcongensis* cells was extracted with Trizol reagent (Invitrogen, Grand Island, NY, USA), digested with DNase I (RNase-free; MBI Fermentas, St. Leon-Roth, Germany) to remove trace DNA, and analyzed on an Agilent Technologies 2100 Bioanalyzer (Agilent, Santa Clara, CA, USA) to verify integrity.

### Real-time PCR

cDNA was synthesized from each RNA sample (1 μg) using the RevertAid H Minus First Strand cDNA Synthesis kit (K1631; MBI Fermentas, St. Leon-Roth, Germany). Real-time PCR assays were conducted in the IQ5 Real-Time PCR Detection System (Bio-Rad Laboratories, Hercules, CA) using the SuperReal PreMix SYBR Green I kit (Toyobo, Osaka, Japan). Primers for real-time PCR are listed in Table S1. Relative expression values of each gene in TTE75 were assigned as 1, and the 16S rRNA gene was used as a reference. All samples were analyzed in triplicate, and the data were represented as means ± SD (standard deviation).

### RNA-seq and analysis of differentially expressed genes (DEGs)

For sequencing of *T. tengcongensis* mRNA, rRNA was removed from total RNA using the Ribo-Zero rRNA Removal kit (Gram-Negative Bacteria, Epicentre Biotechnologies, Madison, WI, USA), then the fragmentation of remaining RNA was carried out using divalent cation fragmentation buffer (New England Biolabs, Ipswich, MA, USA). First-strand cDNA was synthesized using random hexamer primers, and second-strand cDNA was synthesized using a dNTP mixture containing dUTP with DNA polymerase I and RNase H. After adenylation of the 3′ ends of blunt-ended DNA fragments, NEBNext index adaptor oligonucleotides were ligated to the cDNA fragments. The index adaptors were preferentially added to 200-bp cDNA purified using the AMPure XP bead system (Beckman Coulter, Beverly, MA, USA) by PCR with NEB Universal PCR Primers and the index adaptor primer. The second-strand cDNA containing dUTP was digested with the USER enzyme. The first-strand DNA fragments with ligated adaptors on both ends were selectively enriched in a 10-cycle PCR reaction, purified (AMPure XP), and quantified using the Agilent High-Sensitivity DNA assay on the Agilent Bioanalyzer 2100 system. Clustering of the index-coded samples was performed on a cBot Cluster Generation System using the TruSeq PE Cluster Kit v3-cBot-HS (Illumina, CA, USA). After clustering, the libraries were sequenced on an Illumina Hiseq 2000 platform and 100-bp paired-end reads were generated. The raw sequencing data were deposited in the NIH Sequence Read Archive (SRA accession number SRA104298). Clean data were obtained from raw data by removing reads containing adapter, ploy-N and low quality reads. All the downstream analyses were based on the clean data with high quality. Qualified sequences were mapped to the *T. tengcongensis* genome using Bowtie2-2.0.6 [18]. Differential expression analysis for RNA-seq data was performed using the DESeq R package [19]. The resulting *P* values were adjusted using the Benjamini and Hochberg approach for controlling the false discovery rate. Genes with an adjusted *P* value<0.05 were considered significantly differentially expressed.

### Production of *TtecspC* knockout strain

Preparation of medium and genetic transformation for *T. tengcongensis* were performed as described by Liu *et al.* [15]. To construct knock-out plasmid, left homologous arm (204 bp upstream region of *TtecspC*) and left homologous arm (367 bp downstream regions of *TtecspC*) were respectively inserted into plasmid pBK01 to generate the knock-out plasmid pTTCSP. pTTCSP was subsequently introduced into *T. tengcongensis* by natural transformation at three temperatures (50°C, 60°C or 75°C). The transformation mixture was spread on selective medium and then incubated at 50°C, 60°C or 75°C to select transformants.

## Results and Discussion

### *T. tengcongensis* response to rapid temperature downshift

**Figure 1:**
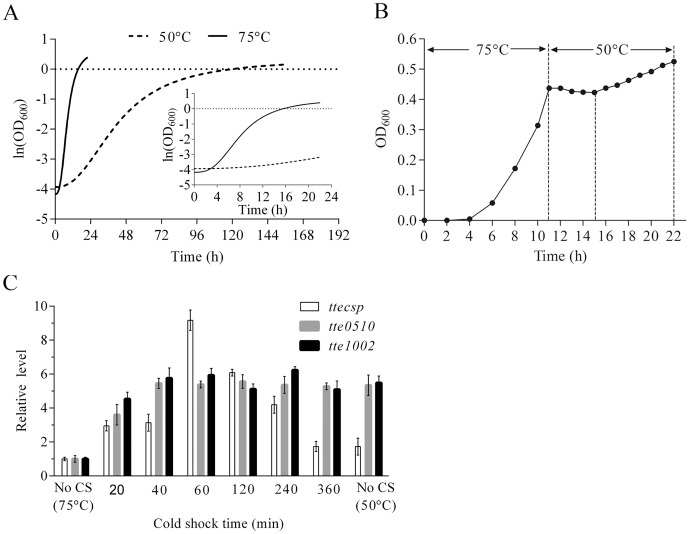
Cold shock response profile of *T. tengcongensis*. A. Growth profiles of separate cultures maintained at 75°C and 50°C. B. Growth profile following rapid temperature shift from 75° to 50°C. C. Transcription profiles of *ttecspC*, *tte0510* and *tte1002* following cold shock. Cells were grown at 75°C to an OD_600_ of 0.6, then cold shocked at 50°C for the indicated times. Control cultures (No CS) were grown constantly at 75° or 50°C. The data are shown as means ± SD (n = 3).

To investigate differences in the growth of *T. tengcongensis* at the optimal temperature (75°C) and the lowest tolerated growth temperature (50°C), growth curves of *T. tengcongensis* were determined at the two temperatures. Although the maximum biomass of *T. tengcongensis* (OD_600_), was not significantly different for the two temperatures (1.3 at 75°C and 1.1 at 50°C), the growth rate was much lower at 50°C than at 75°C (Figure 1A). Although differences in protein expression, which likely lead to the difference in growth rate at low temperature, have been described previously [17], gene expression differences between 75°C and cold shock under 50°C had not been examined prior to this study. As shown in Figure 1B, there was a 4-h lag in *T. tengcongensis* growth upon shift to cold temperature, and then growth resumed at a slower rate. This finding was similar to the classical cold shock response of *E. coli* described by Jones *et al*. [20], and indicates that a cold shock response indeed exists in *T. tengcongensis*.

CSPs play many important roles in bacterial survival under hostile conditions including rapid temperature downshifts [21], and have been shown to accumulate during response to cold shock [10]. As mentioned above, only one *csp* gene (*TtecspC*) has been identified in the *T. tengcongensis* genome. We monitored transcription of *TtecspC* during cold shock at 50°C by real-time PCR. As expected, the temperature downshift resulted in a fluctuation in *TtecspC* transcript levels (Figure 1C). Specifically, *TtecspC* expression constantly rose during the first 60 min of cold shock, reached a peak at 60 min, then gradually decreased over the next 5 h, when it reached the level seen in cells grown constantly at 50°C (Figure 1C).

### RNA-seq and differential gene expression

**Figure 2:**
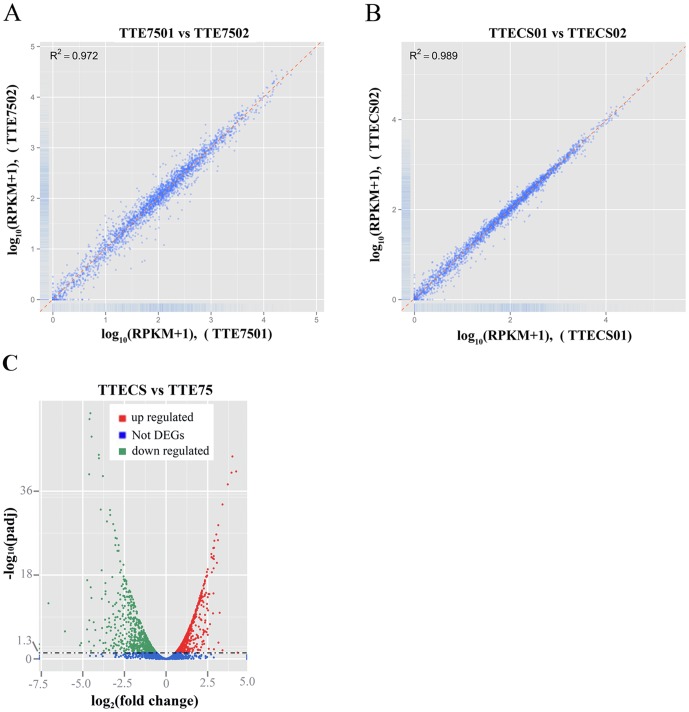
Comparison of gene expression between biological samples with and without cold shock. Scatterplot matrix comparison of gene expression in two biological replicates of TTE75 (A) and TTECS (B). C. Change level of global differential expression genes between TTECS and TTE75.

In order to gain deeper insight into the mechanisms of the *T. tengcongensis* MB4 response to rapid temperature downshifts and the role of *Tte*CspC in survival during cold shock, we performed RNA-seq analysis on transcriptomes from two biological replicates grown at constant 75°C (TTE75) and at 75°C followed by cold shock at 50°C for 1 h (TTECS). Over 99% of all clean reads aligned to coding regions of the *T. tengcongensis* genome (Table 1), and the overall expression levels in the two biological replicates of each group were highly similar to each other (*R* ^2^>0.97; Figures 2A and B), illustrating that our RNA-seq data was of suitable quality for transcriptome analysis.

**Table 1:** Summary of Illumina RNA-seq data.

Sample	Total reads	Total mapped	Clean data* (Gb)	Percent sequence reads mapped
TTE7501	27,936,340	27,711,112	2.8	99.19%
TTE7502	24,982,722	24,826,405	2.5	99.37%
TTECS01	36,140,662	35,829,779	3.6	99.14%
TTECS02	32,537,882	32,297,758	3.3	99.26%

*Clean data were obtained from raw data by removing reads containing adapter, ploy-N and low quality reads.

Between TTECS and TTE75, overall differences in gene expression were observed (Figure 2C). To examine this further, DEGs were identified using the DEseq package [19]. A total of 1295 DEGs were identified, including 652 upregulated and 643 downregulated genes (Table S2). The large number of DEGs suggests that temperature downshifts have global effects on *T. tengcongensis*.

The 20 most highly expressed genes in TTESC and TTE75 are listed in Table S3. Twelve of these genes in TTE75 encode proteins detected in the *T. tengcongensis* proteome (AhpC, TTE1894, TTE0106, HimA, MalE, SpoVG2, TTE2480, TTE0932, SpoVS3, RpmE, Fer, and TTE1251) [22], indicating a good accordance between gene transcription and protein translation data and further validating our RNA-seq analysis.

The top 10 upregulated and ten most downregulated *T. tengcongensis* genes under cold shock are listed in Table 2. Among the most upregulated genes, five were predicted genes of unknown function, and therefore their roles in cold shock response need to be further investigated. One of the remaining five genes encodes *Tte*CspC, a member of the *Eco*CspA family considered important in cold shock response. Another encodes Met17, an *O*-acetylhomoserine sulfhydrylase involved in synthesis of sulfur amino acid. The upregulation of Met17 during cold shock may reflect a greater need for products of the Met17-related biosynthetic pathway, such as cysteine, methionine, and glutathione, to cope with low-temperature stress, as has been shown for the response to oxidative stress [23]–[25]. Two other upregulated genes (*mltE2*, *lytE2*) encode for lytic murein transglycosylase MltE and putative peptidoglycan endopeptidase LytE, both of which are involved in metabolism of peptidoglycan, a critical structural component of cell walls and thus a determinant of cell shape and viability. This might indicate that remodulation of cell wall by altering content of peptidoglycan was also required for the acclimation of low temperature, and the similar cases were reported previously [26], [27]. Additionally, *nhaC* encoding Na^+^/H^+^ antiporter probably participated in cold shock response due to the significant increase of its transcript level during cold shock.

**Table 2:** Top ten up- and downregulated *T. tengcongensis* genes after cold shock at 50°C for 1 h.

Rank	Gene	Log_2_(fold change)	Annotation
1↑	*tte1002*	4.2	Hypothetical protein
2↑	*cspC*	4.0	Cold shock protein
3↑	*met17*	3.9	*O*-Acetylhomoserine sulfhydrylase
4↑	*mltE2*	3.7	Lytic murein transglycosylase
5↑	*tte0510*	3.4	Hypothetical protein
6↑	*tte1291*	3.2	Hypothetical protein
7↑	*novel012* *	3.1	Transposase domain protein
8↑	*tte0165*	3.1	Hypothetical protein
9↑	*lytE4*	3.1	LysM repeat-containing protein
10↑	*nhaC*	3.1	Na^+^/H^+^ antiporter
1↓	*tte2770*	−7.1	astB/chuR-related protein
2↓	*mdlB12*	−6.1	ATPase component
3↓	*tte2654*	−5.2	Hypothetical protein
4↓	*tar5*	−5.1	Methyl-accepting chemotaxis protein
5↓	*tte2745*	−4.7	Hypothetical protein
6↓	*pspF4*	−4.7	Transcriptional regulator
7↓	*tte0328*	−4.6	Thioredoxin-like protein Tlp
8↓	*tte0792*	−4.6	Hypothetical protein
9↓	*rbsB4*	−4.6	Periplasmic sugar-binding protein
10↓	*galT*	−4.6	Uridylyltransferase

↑: up-regulated;

↓: down-regulated;

* novel transcript detected by RNA-Seq.

**Figure 3:**
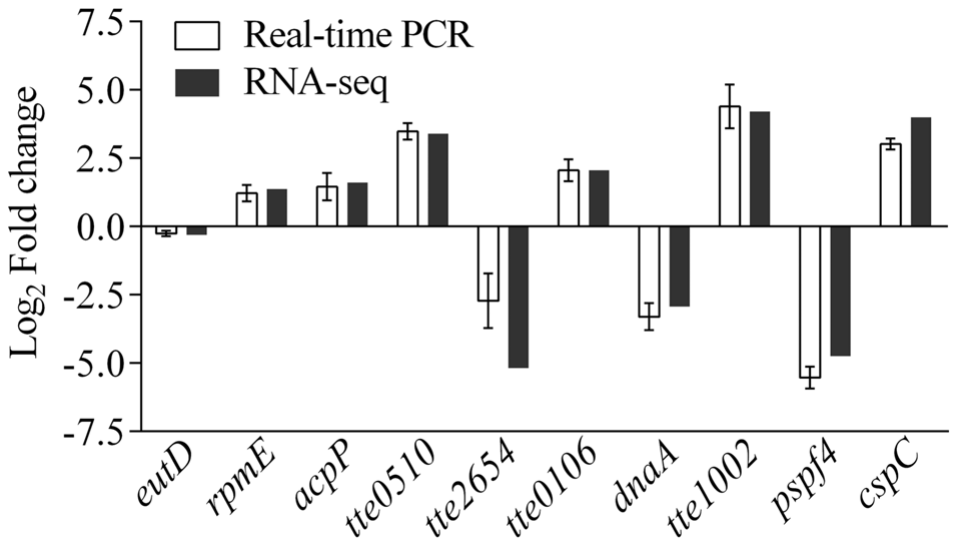
Validation of differentially expressed genes by real-time PCR. Comparison of RNA-seq and real-time PCR measures of changes in expression of *eutD* (encoding phosphotransacetylase), *rpmE* (50S ribosomal protein L31), *acpP* (acyl carrier protein), *tte0510* (hypothetical protein), *tte2654* (hypothetical protein), *tte0106* (hypothetical protein), *dnaA* (chromosome replication initiator DnaA), *tte1002* (hypothetical protein), *pspf4* (a transcriptional regulator), and *cspC* (cold shock protein). All data are shown as means ± SD (n = 3).

To more thoroughly examine the expression of genes identified in the RNA-seq analysis, we performed real-time PCR on three of the most highly expressed genes (*rpmE*, *acpP* and *tte0106*), three of the most upregulated genes (*tte1002*, *tte0510* and *TtecspC*), two of the most downregulated genes (*tte2654* and *pspf4*), one moderately repressed gene (*dnaA*), and one gene that was not differentially expressed (*eutD*). The expression profiles of the selected genes showed the same tendencies by real-time PCR as detected by RNA-seq, with only minor quantitative differences between the two methods (Figure 3).

### Gene Ontology (GO) annotation and KEGG pathway mapping of DEGs

**Figure 4:**
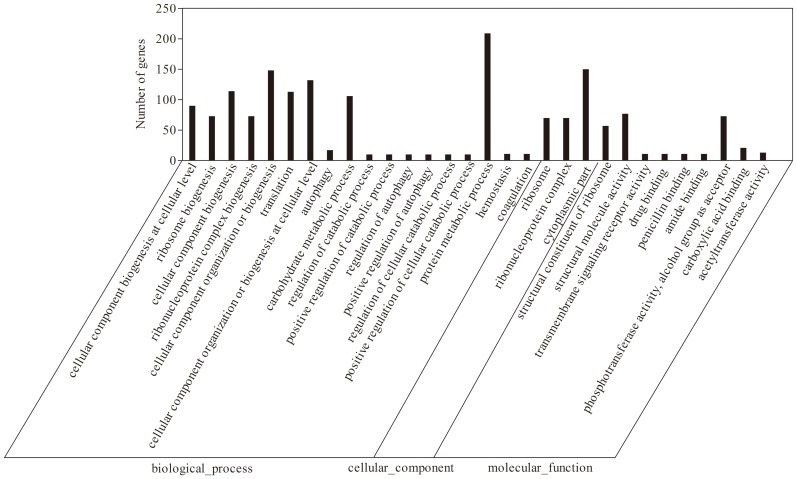
GO analysis of differentially expressed genes in *T. tengcongensis* with and without cold shock.

The DEGs were assigned to 30 functional groups by enrichment analysis of GO assignments. In the three main GO categories of biological process, cellular component, and molecular function, the most dominant subcategories were “protein metabolic process”, “cytoplasmic part”, and “structural molecule activity”, respectively (Figure 4). In addition, “cellular component organization or biogenesis”, “ribonucleoprotein complex”, and “carboxylic acid binding” were also well represented.

**Figure 5:**
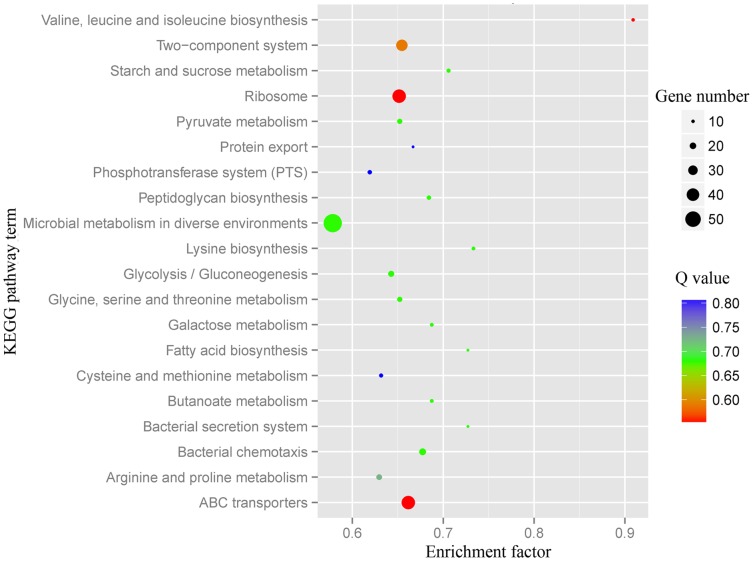
KEGG pathway enrichment analysis of differentially expressed genes between TTCS and TTE75.

The biological functions associated with DEGs were further analyzed in terms of enriched Kyoto Encyclopedia of Genes and Genomes (KEGG) pathways [28], and a total of 20 pathways were predicted (Figure 5). Among them, “microbial metabolism in diverse environments”, “ABC transporters”, “ribosome”, and “two-component system” were the most highly represented categories.

### Biological roles of *Tte*CspC

mRNA tends to form secondary structures, especially at low temperature, that decrease the rate of protein synthesis [21]. Some members of the CSP family, such as *Eco*CspA and *B. subtilis* CspB, are induced by cold shock and function as RNA chaperones to prevent the formation of mRNA secondary structures, therefore facilitating efficient ribosomal binding, protein elongation, and translation termination [29]. *Eco*CspA also significantly accumulates on entry into log growth phase [30], and *Staphylococcus aureus* CspC is strongly induced by some antibiotics and toxic chemicals [31]. Therefore, CSPs are essential to survival not only during cold shock, but also when cells encounter other stressors. Recently, CSPs in *Listeria monocytogenes* were reported to regulate the production of the pore-forming cytolysin listeriolysin, probably by preventing degradation of the corresponding transcripts [32]. Overall, CSPs act as multifunctional nucleic acid−binding proteins to regulate a wide range of gene expression responses including, but not limited to, cold shock in bacteria. As mentioned above, a single Csp gene, *TtecspC*, was harbored in *T. tengcongensis*, therefore we speculated that *Tte*CspC was responsible for not only cold shock response but also survival of *T. tengcongensis* at optimal growth temperature.

As shown in Table S2, *TtecspC* was the seventh most abundantly expressed gene at 75°C, suggesting that *Tte*CspC is required for *T. tengcongensis* survival even at optimal growth temperature. This was further confirmed by the fact that our efforts to disrupt *TtecspC* by gene knockout were unsuccessful. Under cold shock, *TtecspC* was the most highly expressed gene (Table S3) and was the second-most upregulated gene (15.8 fold; Table 2), indicating an equally important role of *Tte*CspC in the cold shock response of *T. tengcongensis*.

Taken together, these results suggest that the cold shock response in *T. tengcongensis* is different in some central aspects from that in another thermophile, *T. thermophilus*, at least in terms of CSPs. In contrast to the single *Tte*CspC in *T. tengcongensis*, *T. thermophilus* has two Csps: one that mainly contributes to translational control under optimal growth conditions (Csp1) [33], and another that is mainly responsible for the cold shock response (Csp2) [2]. Identifying the genes regulated by *Tte*CspC and the corresponding mechanisms of regulation will clarify this further.

Interestingly, although *TtecspC* was highly expressed at both high and low temperatures, *Tte*CspC has not been detected previously in the *T. tengcongensis* proteome [17], [22]. This might be because some aspects of proteome preparation, such as pH ranges and molecular weights isolated from two-dimensional gel electrophoresis, were inappropriate for isolation of a small protein like *Tte*CspC (66 amino acids).

### DEGs involved in transcriptional regulation

Changes in the expression of transcription factors, especially master regulators, can modify global transcriptional regulatory networks, which in some cases is essential for adaptation to new environmental conditions. Analysis of the *T. tengcongensis* genome revealed 70 potential transcriptional regulator genes (∼2.7% of total protein-coding genes). Approximately half of these transcriptional regulators (51.4%) were expressed at similar levels in TTECS and TTE75 and 48.6% were differentially expressed, including 8.6% that were upregulated and 40% that were downregulated. The fact that such a large proportion of transcriptional regulators were differentially expressed indicates that cold shock exerts global effects on gene expression in *T. tengcongensis*. The most elevated transcriptional regulator was AbrB, an ambiactive regulator of genes related to the transition between vegetative growth and the onset of stationary phase and sporulation in *B. subtilis* [34]. Substantially elevated AbrB suggests that *T. tengcongensis* may respond to cold shock by entering into stationary phase and sporulation, which was further supported by the fact that many sporulation-related genes were upregulated during cold shock. Among the downregulated transcriptional regulators, PspF4 was the most repressed (28.7 fold), suggesting that it is not essential for cold shock adaptation in *T. tengcongensis*, similar to its homolog in *B. subtilis*, LevR [35].

### DEGs involved in DNA replication, recombination, and repair

Three inextricably linked processes–DNA replication, recombination, and repair–preserve the fidelity and integrity of the genome [36]. These processes can be modulated by changes in environmental temperatures [37]. Thus, we examined the expression of genes related to these processes during cold shock in *T. tengcongensis*.

Representative upregulated factors associated with DNA replication included genes encoding TopA, the α subunit and γδ complex of DNA polymerase III, DnaN, RNase H, RecN/G, HolB, and Ssb2/Ssb3, most of which are known to be low-temperature inducible [38]. Interestingly, *dnaA*, which is induced in *E. coli* and *Moraxella catarrhalis* at low temperature [38], [39], was repressed in our study, illustrating a distinctive cold shock response in *T. tengcongensis*.

Additionally, many DNA recombination–related genes were induced by exposure to cold, such as genes encoding the filament formation–related RecJ and Ssb2/Ssb3, the UvrA/B/C proteins involved in ATP-dependent branch migration of the Holliday junction, the DNA strand exchange inhibitor (TTE1685), the site-specific integrase-resolvase (TTE2162) and the site-specific tyrosine recombinase (XerC). Similarly, factors involved in DNA repair were also induced by cold, such as genes for the DNA repair photolyase (SplB), the DNA repair ATPase (SbcC), and the DNA repair protein (RadC). In summary, low temperature promoted DNA recombination and repair processes in *T. tengcongensis*, which is consistent with the observation that cold shock increases the rate of recombination in *Drosophila melanogaster* [40].

### DEGs involved in transcription, translation, and posttranslational processes

Cold shock induces stabilization of mRNA secondary structures, which can further lead to reduced translational efficiency, slow or inefficient protein folding, and reassembly of ribosomes to function properly at low temperature. The activity of DNA-dependent RNA polymerase can be modified by binding interchangeable σ factors to transcribe genes required for specific environmental conditions or stressors [41]. During cold shock, we found that the RNA polymerase core enzyme genes *rpoA*, *rpoB*, and *rpoZ* were upregulated. The expression of genes for the σ factors RpoE6 (σ70), RpoE7 (SigX, which regulates cell envelope modification in *B. subtilis*), RpoD2 (SigG), and RpoD3 (sporulation σ factor SigE) were also upregulated whereas genes for σ factors RpoE3, RpoE4, RpoN (σ54 homologs), and FliA (SigI) were moderately repressed. Therefore, it appears that cold shock caused changes in the expression of components of RNA polymerase that promote the association of the polymerase with different σ factors to transcribe specific cold-responsive genes, such as those involved in sporulation. In addition, several other transcription factor genes were induced by low temperature, including those encoding transcription elongation factor NusA, transcription termination factor Rho, and transcription antitermination factors NusG and NusB.

It has been proposed that ribosomes may act as the sensor for thermal stress and that they may reassemble to promote synthesis of specific proteins for adaptation to various temperatures [42]. This appears to be the case in *T. tengcongensis*, because we observed that expression of several genes for translation factors and components specifically associated with the ribosome were elevated during cold shock. Genes for 43 ribosomal proteins were upregulated, some of which are already known to be cold-inducible (S6, L7/L12, L27, L31, and S15) [43], [44]. Genes for InfA and InfB, two initiation factor homologs, were induced two-fold by cold shock, suggesting that translation elongation may also be altered at low temperatures in *T. tengcongensis*. Expression of seryl-, cysteinyl-, methionyl-, leucyl-, prolyl-, and phenylalanyl-tRNA synthetase genes was elevated under cold shock, whereas expression of alanyl- and lysyl-tRNA synthetase genes was decreased. Such changes in the relative proportions of specific tRNA synthetases are consistent with the widespread changes in protein synthesis associated with cold shock response. Finally, some genes encoding factors involved in posttranslational processes were affected by cold shock. For example, several genes for heat-shock proteins (GrpE, DnaK, GroES, and GroEL) were repressed by >3 fold, whereas gene expression for the cold-induced molecular chaperone caseinolytic protease (Clps) was elevated by 2.2 fold.

### DEGs involved in cell wall and membrane structures

Cell walls are the major structural component of cells and are the primary protection against environmental stresses in many organisms. Peptidoglycan biosynthesis is a critical process in the formation of bacterial cell walls. Several genes involved in cell envelope and membrane biogenesis were differentially expressed when *T. tengcongensis* was shifted from 75° to 50°C. In particular, the gene encoding RfaG2, which is involved in synthesis of lipopolysaccharides, was downregulated by 16.4 fold, which was similar to what has been observed in *Shewanella oneidensis* MR-1 at low temperature [45]. On the other hand, two observations suggest that *N*-acetylglucosamine (GlcNAc) content in the cell walls of *T. tengcongensis* might be enhanced by cold shock. First, gene expression for the transcriptional regulator NagC, which negatively regulates *N*-acetylglucosamine (GlcNAc) utilization, was upregulated by 3.2 fold. Second, 13 genes involved in peptidoglycan synthesis were induced by low temperature. These findings strongly suggest that cell wall remodeling through increased GlcNAc content and peptidoglycan modification might be essential to the adaptation to rapid temperature downshifts.

The fluidity of cell membranes can be enhanced by increasing branched-chain fatty acid content [46]. The branched-chain amino acids isoleucine, valine, and leucine are the most abundant amino acids and form the hydrophobic core of proteins [47], and are also precursors for biosynthesis of iso- and anteiso-branched-chain fatty acids [48]. In *T. tengcongensis*, the gene for PaaI, a negative regulator of genes involved in fatty acid and phospholipid biosynthesis, was upregulated by 2.9 fold after cold shock, which could result in decreased fatty acid biosynthesis. On the other hand, the expression of some genes involved in synthesis of branched-chain amino acids (*leuA/B/C/D* and *ilvB/C/D/E/H*) and in fatty acid synthesis (*fabH/G/K*) was also elevated. These findings suggest that cold adaptation in *T. tengcongensis* may involve an increase in branched-chain fatty acids combined with a decrease in straight-chain saturated fatty acids to ensure the fluidity of the cell membrane. Additionally, glycerophospholipid metabolism also appeared to be enhanced by cold shock in *T. tengcongensis* based on the increased expression of several enzymes in this pathway. We therefore propose that upregulation of branched-fatty acid biosynthesis combined with increased incorporation of these fatty acids into glycerophospholipids provides the necessary fluidity to cell membranes at low temperatures.

### DEGs involved in flagellar formation and motility and sporulation

Flagellar biosynthesis and motility play crucial roles in bacterial responses to various environmental stresses [49], [50], and flagellar-motility gene transcription is temperature dependent [50]. Although functional flagella were not observed in *T. tengcongensis* under the culture conditions described by Xue *et al*. [13], this bacterium possesses 29 essential genes for flagellar biogenesis and nearly all the genes for the chemotaxis signaling pathways [14]. Interestingly, 14 DEGs involved in flagellar assembly were moderately induced (∼1.6–4 fold) in *T. tengcongensis* at low temperature, consistent with the observation that induction of genes involved in flagellar synthesis and chemotaxis is one of the most prominent responses to cold in *Yersinia enterocolitica* [51]. It is notable that the expression of the four flagellar-related genes *fliG*, *fliM*, *flgC*, and *flgB* was detected by RNA-seq even though the proteins were not detected in the proteome of *T. tengcongensis* [22].

Endospore formation is a critical response of sporulating bacteria to environmental stresses such as starvation, exposure to ethanol, and exposure to high or low temperature [52], [53]. There are 23 sporulation-related coding sequences in the *T. tengcongensis* genome [14]. Under low temperature stress, we found that expression of several of these genes increased. As mentioned earlier, the stationary phase/sporulation-related transcriptional regulator AbrB was induced. In addition, expression levels of the genes for sporulation-related σ factors, such as SigG and SigE, and other sporulation-related proteins, including SpoII D, SpoIIGA, TTE0969 and TTE162*5*, were elevated. These observations suggest that *T. tengcongensis* may withstand low temperature in part by undergoing sporulation.

### DEGs involved in carbon metabolism and energy production and conversion

*T. tengcongensis* carries genes for all known proteins involved in the glycolysis and pentose phosphate pathways, indicating *T. tengcongensis* can utilize sugars as a principal energy and carbon source [14]. Expression of the genes for three key glycolytic enzymes (glucokinase, 6-phosphofructokinase, and pyruvate kinase) and for triosephosphate isomerase was elevated during cold shock. *T. tengcongensis* possesses only about half of the known genes involved in the tricarboxylic acid cycle [14]; among them, only the gene for 2-oxoglutarate ferredoxin oxidoreductase was upregulated under cold shock. Additionally, several genes encoding proteins related to electron transport were affected under cold shock. Specifically, NADH dehydrogenase-related genes such as *nuoG* and *nuoF* were elevated (∼4 fold), whereas *nuoL*, *nuoH*, and *nuoB* were downregulated (∼2 fold), and genes encoding ferredoxin, dihydrolipoamide acyltransferase, rubredoxin, thioredoxin, and thioredoxin reductase were upregulated (∼2–7 fold). These observations suggest that *T. tengcongensis* has higher energy demands at low temperature, perhaps to fuel the increased production of specific proteins required to respond to cold shock.

### DEGs involved in the ABC transporter and bacterial secretion systems

Among the numerous ABC transporter genes identified in the *T. tengcongensis* genome, only those for biotin and phosphate transport were upregulated, whereas those for multiple sugar, cyclodextrin, oligopeptide, iron complex, zinc, cobalt/nickel, Na^+^, and antibiotic transport were significantly downregulated. Proteins are primarily secreted via the signal recognition particle (SRP) pathway in *T. tengcongensis*. We found that genes for several components of the SRP complex, including *secA/D/E/F*, s*ecY*, *yajC*, and *ftsY* were upregulated during cold shock.

### Induction of signal transduction

Signal transduction, a principal mechanism by which cells respond to various environmental cues, is mostly conducted through two-component regulatory systems in prokaryotes. We identified 40 two-component regulatory system proteins (excluding those involved in chemotaxis) in the *T. tengcongensis* genome using the Microbial Signal Transduction database (MiST2.2; http://mistdb.com/bacterial_genomes/summary/414). Among the 19 kinase genes identified, two (*tte0886* and *tte1682*) were upregulated and five (*tte0287*, *tte1049*, *tte1050*, *tte2344*, and *tte2392*) were downregulated. Among 21 response regulator genes, nine were differentially expressed, including five that were upregulated and four that were downregulated. Among the 12 signal transduction–related operons, three were upregulated (*lytT* and *baeS2*, *citB* and *baeS8*, and *araC* and *lytS2*). Expression of the *citB* and *baeS8* operon was elevated >2 fold, indicating that signal transduction related to this operon is likely involved in the general cold shock response.

Among the 23 genes for chemotaxis-related proteins identified in the *T. tengcongensis* genome, genes for eight methyl-accepting chemotaxis proteins, chemotaxis protein histidine kinase (CheA), chemotaxis signal transduction protein (CheW), and methyltransferases (CheR and CheB) were downregulated. In contrast, expression of genes for chemotaxis response regulator CheY and the flagellar motor switch protein FliN/FliG were moderately elevated.

### Induction of novel genes and genes with unknown function

The gene-product functions of 31.8% of the predicted coding sequences in *T. tengcongensis* are unknown. Among the top 20 significantly differentially expressed genes we identified, seven encoded hypothetical proteins (Table 2). These hypothetical proteins may play novel physiological roles in *T. tengcongensis* cold adaptation and acclimation. To better understand the effects of cold shock on these hypothetical proteins, the expression profiles of two representative genes, *tte1002* and *tte0510*, were examined following cold shock using real-time PCR. Within 20 min of temperature downshift, both *tte1002* and *tte0510* were induced to a much higher degree even than *TtecspC* (Figure 1C). Moreover, unlike *TtecspC*, expression of these two genes remained elevated during the entire cold shock period, indicating that they are likely required for both the initial response to cold shock and longer-term survival at low temperature. Finally, some potentially novel genes were identified in this study, suggesting that these genes are expressed only under cold shock. These are listed in Table S4. At the present time, the mechanisms for regulation of these and other cold-modulated genes is not known, but may include mediation by *Tte*CspC or auto-regulation. Given their significant differential expression, these genes are candidates for further investigation of regulatory mechanisms underlying cold shock in *T. tengcongensis*.

### Natural competence

Natural competence, one form of horizontal DNA exchange, involves the binding and uptake of extracellular DNA [54], [55], and is an adaptation mechanism in response to external factors such as pheromone density (quorum sensing) or stringent nutritional conditions [56]. Natural competence involves three steps: (i) DNA binding, probably via type IV pili, (ii) DNA cleavage and transport of single-stranded DNA by ComEA and ComEC, and (iii) DNA recombination by CinA-localized RecA or DNA replication (for plasmid DNA) [57]. The DNA uptake system in *B. subtilis* is the best characterized, consisting of three proteins, ComEA, ComEC, and ComFA [57].

Previously, we reported that *T. tengcongensis* is naturally competent [15]. Transformation frequency is dependent on the physiological phase of the cells, with the highest transformation efficiency occurring during the early log-growth phase and the lowest occurring during stationary phase; additionally, transformation frequency is also highly dependent on temperature, with transformation efficiency at 50°C being markedly lower than that at 75°C [15]. In *T. tengcongensis*, no putative type IV pilus locus is evident in the genome, but several loci (*tte1262/1263/1267–1270*) are thought to be related to type IV pilus assembly. Among these, only *tte1263*, annotated as pili biogenesis protein PilT-like ATPase, was differentially expressed (induced) in our analyses. The fact that expression of genes involved in DNA recombination was elevated at low temperature suggests that DNA uptake should be the rate-limiting step of natural competence in *T. tengcongensis*. We speculated that low DNA uptake efficiency would arise primarily from reduced expression of DNA transporters, and this hypothesis was supported by this study. First, low temperature led to reduced expression of the genes for ComEA (TTE0925) and ComEC2 (TTE2430), two proteins required for DNA transport into the cytosol and essential for natural competence in *Thermoanaerobacterium saccharolyticum*, although it increased expression of genes encoding CinA and RecA, which are important but not essential for natural competence [58]. Second, energy production during cold shock is generally low, which may limit the relatively high energy-consuming process of DNA uptake.

## Conclusions

Cold shock has a profound impact on cell growth by influencing ribosomal synthesis, cytoplasmic membrane composition and fluidity, protein synthesis, and solute uptake. In this analysis, cold shock in *T. tengcongensis* caused global alterations in gene expression that led to multiple physiological changes. This transcriptome analysis identified numerous genes involved in the *T. tengcongensis* cold shock response, demonstrating effects on diverse processes, including DNA replication, recombination, and repair; transcriptional and translational regulation of gene expression; biogenesis of cell walls, cell membranes, and flagella; maintenance of cell membrane fluidity, carbohydrate and energy metabolism; and natural competence. A number of novel genes, expressed only under reduced temperature conditions, were also identified. Moreover, the cold shock protein *Tte*CspC and/or other cold-inducible proteins appear to function as molecular chaperones to facilitate specific protein synthesis and regulate these various molecular, cellular, and physiological changes, allowing cells to adapt to temperature downshift.

*Thermoanaerobacter* strains can survive in similar environments and have similar properties. Recently, another important property, natural competence, was revealed in several *Thermoanaerobacter* strains including *T. tengcongensis* [15], [58]. We thereby speculate that it might be common that *Thermoanaerobacter* strains harbor a single *csp*-like gene that responds to cold shock. However, due to the limitations associated with the use of only a single strain, we have not much evidence to verify it. Further studies will be conducted on other *Thermoanaerobacter* strains to determine whether these strains have similar mechanisms of cold shock responses as *T. tengcongensis*. These data in this study add to our understanding of the mechanisms of cold shock response in *T. tengcongensis* and also shed light on the adaptation mechanisms of other *Thermoanaerobacter* bacteria under temperature downshift.

## Supporting Information

Table S1 **Primers used for real-time PCR.**
(DOCX)

Table S2 **Differentially expressed genes according to DESeq analysis.**
(XLSX)

Table S3 **Twenty most highly transcribed genes in TTECS and TTE75.**
(DOCX)

Table S4 **Novel genes in TTECS identified by RNA-seq.**
(XLSX)
