# Supplementary material for: RNA-Seq-Based Analysis of Cold Shock Response in Thermoanaerobacter tengcongensis, a Bacterium Harboring a Single Cold Shock Protein Encoding Gene
Source: PLoS One. 2014 Mar 25;9(3):e93289. doi: 10.1371/journal.pone.0093289 (PMC3965559; doi:10.1371/journal.pone.0093289)
Supplement: Table S1 — Primers used for real-time PCR. (DOCX) [file pone.0093289.s001.docx]

**Table S1.** Primers used for real-time PCR

| Name | Sequence (5′ to 3′) |
| --- | --- |
| *eutD*-F | CATTATGAAGGCACTAGAAACCAC |
| *eutD*-R | AAGGGATGACAGAGGAAGAAGC |
| *rpmE*-F | CGATGCTGTTGTGAAATGTGC |
| *rpmE*-R | CTCTCGACTCTTCCACCTGTATCTAC |
| *acpP*-F | GTCTTTATTTTTTCGGCGTCTTC |
| *acpP*-R | GAAAAAGTGAGAAACATCATTGCTG |
| *tte0510*-F | GGAACAGCAATTTTATGCAGAGA |
| *tte0510*-F | ATGGTGGTTATGGCTTCTCCTC |
| *tte2654*-F | ACCATCCTTTCCTTATTTCCTCA |
| *tte2654*-R | CGACTCATTTCATTAGCACTCCA |
| *tte0106-*F | GCAGAAAATGCATGCTGGTTC |
| *tte0106-*R | AGTAGATGAGCTGGGAAGGGTAG |
| *dnaA*-F | CACTTAATGCACGCTATAGGACAC |
| *dnaA*-R | CTTCGTTTTTATCGTCCTTGATG |
| *tte1002*-F | CCAGGCATAGTTTTACCTTCAGAG |
| *tte1002*-R | CAAATCTCCCTGCGCTACGA |
| *pspf4*-F | CAATCCTCAGCTTCTCCTTTCTC |
| *pspf4*-R | CGGAGGAAGGCGATGAAC |
| *cspC*-F | CTTTAGCCGCCTGAACTACCTC |
| *cspC*-R | TTGAGAGAGAGGATGGCACAGAC |
| 16S rRNA-F | CAAGGCTGAAACTCAAAGGAAT |
| 16S rRNA-R | GACTTAACCCAACATCTCACGAC |
