# Supplementary material for: RNA-Seq-Based Analysis of Cold Shock Response in Thermoanaerobacter tengcongensis, a Bacterium Harboring a Single Cold Shock Protein Encoding Gene
Source: PLoS One. 2014 Mar 25;9(3):e93289. doi: 10.1371/journal.pone.0093289 (PMC3965559; doi:10.1371/journal.pone.0093289)
Supplement: Table S3 — Twenty most highly transcribed genes in TTECS and TTE75. (DOCX) [file pone.0093289.s003.docx]

|  | **TTECS** | | **TTE75** | |
| --- | --- | --- | --- | --- |
| Rank | Gene | Product | Gene | Product |
| 1 | *cspC* | Cold shock protein | *ahpC* | Peroxiredoxin |
| 2 | *tte0106* | Hypothetical protein | *tte1894* | Hypothetical protein |
| 3 | *tte1251* | Hypothetical protein | *tte0106* | Transcriptional regulator AbrB |
| 4 | *tte0510* | Hypothetical protein | *himA* | Nucleoid DNA-binding protein |
| 5 | *ahpC* | Peroxiredoxin | *malE* | Maltose ABC transporter substrate-binding protein |
| 6 | *rpmE* | 50S ribosomal protein L31 | *spoVG2* | Regulatory protein SpoVG |
| 7 | *acpP* | Acyl carrier protein | *cspC* | Cold shock protein |
| 8 | *spoVG2* | Regulatory protein SpoVG | *tte2480* | Major membrane immunogen precursor |
| 9 | *tte1894* | Hypothetical protein | *tte0932* | Ferredoxin 3 |
| 10 | *rpmB* | 50S ribosomal protein L28 | *spoVS3* | Stage V sporulation protein S |
| 11 | *fer* | Ferredoxin 1 | *rpmE* | 50S ribosomal protein L31 |
| 12 | *tte2382* | Predicted membrane protein | *novel00038* | --^*^ |
| 13 | *tte2561* | Hypothetical protein | *novel00013* | --^*^ |
| 14 | *tte0037* | Cytosine/adenosine deaminase | *fer* | Ferredoxin 1 |
| 15 | *tte2230* | Hypothetical protein | *rpsT* | 30S ribosomal protein S20 |
| 16 | *rpsR* | 30S ribosomal protein S18 | *tte0037* | Cytosine/adenosine deaminase |
| 17 | *novel00033* | --^*^ | *tte2561* | Hypothetical protein |
| 18 | *tte0521* | Hypothetical protein | *tte1251* | Hypothetical protein |
| 19 | *rplA* | 50S ribosomal protein L1 | *tte0272* | Hypothetical protein |
| 20 | *novel00014* | --^*^ | *tte0521* | Hypothetical protein |

**Table S3.** Twenty most highly transcribed genes in TTECS and TTE75.

^*^ Not annotated.
